# From Cultured Vascular Cells to Vessels: The Cellular and Molecular Basis of Vascular Dysfunction in Space

**DOI:** 10.3389/fbioe.2022.862059

**Published:** 2022-04-05

**Authors:** Laura Locatelli, Sara Castiglioni, Jeanette A. M. Maier

**Affiliations:** ^1^ Department of Biomedical and Clinical Sciences L. Sacco, Università di Milano, Milano, Italy; ^2^ Interdisciplinary Centre for Nanostructured Materials and Interfaces (CIMaINa), Università di Milano, Milan, Italy

**Keywords:** spaceflight, microgravity, vessel, endothelial cells, vascular smooth muscle cells

## Abstract

Life evolved on this planet under the pull of gravity, shielded from radiation by the magnetosphere and shaped by circadian rhythms due to Earth’s rotation on its axis. Once living beings leave such a protective environment, adaptive responses are activated to grant survival. In view of long manned mission out of Earth’s orbit, it is relevant to understand how humans adapt to space and if the responses activated might reveal detrimental in the long run. Here we review present knowledge about the effects on the vessels of various extraterrestrial factors on humans as well as *in vivo* and *in vitro* experimental models. It emerges that the vasculature activates complex adaptive responses finalized to supply oxygen and nutrients to all the tissues and to remove metabolic waste and carbon dioxide. Most studies point to oxidative stress and mitochondrial dysfunction as mediators of vascular alterations in space. Unraveling the cellular and molecular mechanisms involved in these adaptive processes might offer hints to design proper and personalized countermeasures to predict a safe future in space.

## Introduction

The peripheral vascular system consists of an intricate network of blood vessels that maintains tissue homeostasis. Vessels not only act as a transport conduit system that delivers oxygen and nutrients to the tissues while removing wastes, but also importantly contribute to tissue morphogenesis, development, metabolism, inflammation, and wound healing (Shimokawa and Satoh, 2014; Ramasamy et al., 2015; Tucker et al., 2022). In spite of specific regional characteristics depending on the organ they perfuse, arteries, capillaries and veins share common histological features. Arteries, which are crucial for nourishing tissues, and veins, which transport wastes away from tissues, are composed of three layers, the tunica intima, media and adventitia (Pugsley and Tabrizchi, 2000). The tunica intima is the innermost layer, and consists of endothelial cells (EC) on a basal lamina. The tunica media, composed by smooth muscle cells and elastin, is better organized in arteries than in veins, while the tunica adventitia is the outermost layer and is composed of connective tissue containing small vessels, lymphatics and nerve plexi (Pugsley and Tabrizchi, 2000; Tucker et al., 2022). The capillary network lies between the arteries and the veins, and consists of endothelial cells on a basal membrane in which pericytes are embedded. It is in the capillary bed that nutrients, metabolites, water and gases are exchanged between the tissues and the blood. Vascular homeostasis is the result of the complex interplay among the various cell types physiologically localized in the vessels -endothelial and smooth muscle cells, pericytes-, the extracellular matrix components, the autonomic nervous system and the eventually present inflammatory cells. Here we summarize recent advancements on the effects of spaceflight on the vasculature, taking into account the plethora of challenges that vessels face in space, such as microgravity, alterations of circadian rhythm, inactivity and psychological stress.

By searching PubMed, Google Scholar, the NASA and ESA websites from 2000 to date (February 2022), we review existing evidence from experimental and clinical studies about the effects of space flight on the vasculature. We selected only papers written in English and covering *in vitro*, animal and human studies. The keywords included “microgravity”, “spaceflight”, “radiation”, “vessels”, “endothelial cells”, “vascular smooth muscle cells”, “circadian rhythm”. We will initially describe how vessels react in space and then we will try to get insights into the cellular and molecular mechanisms involved by drawing on data obtained using vascular cells in culture. Understanding the basis of vascular alterations in space might lead to countermeasures that preserve vascular function and mitigate most of medical issues associated with spaceflight ensuring proper peripheral supply of metabolites and removal of wastes.

## Vascular Stress in Space: The Effects of Microgravity, Radiation, Emotional Strain, Sedentary Life-Style and Altered Circadian Cycles on the Vasculature

Space is an extreme environment that activates a series of adaptive responses to various extraterrestrial factors, from microgravity to space radiation, from altered circadian rhythms and style of life to psychological stress. Life developed and evolved on our planet under the pull of gravity and all living beings are adapted to function optimally at 1 g. Opposing gravity is very demanding for humans that maintain an erect posture in line with the gravitational pull. Indeed, terrestrial bipedalism required the reallocation of blood supply to preserve a steady mean arterial pressure and grant an adequate perfusion to the brain. One of the earliest events astronauts experience is the shift of fluids from the lower limbs to the head, which results in the loss of hydrostatic pressure gradients (Patel, 2020). Rapidly, fluid redistribution triggers atrial natriuretic factor release, the inhibition of the renin-angiotensin-aldosterone system and autonomic neural responses (Patel, 2020). Because of the increased hydrostatic pressure above the heart and the gradual impairment of lymphatic drainage, and also through the action of locally released vasopermeabilizing factors, fluids shift from the intravascular to the interstitial compartment, thus explaining the facial edema of astronauts (Baran et al., 2022). Over time neurovegetative and endocrine responses to microgravity intervene to reach a novel homeostasis. After months in space, the levels of circulating angiotensin II and catecholamines as well as blood insulin increase (Hughson et al., 2016; Hughson et al., 2018). In parallel, carotids and femoral arteries show a thicker tunica media, which leads to enhanced stiffness (Hughson et al., 2016; Hughson et al., 2018; Norsk, 2020). Notably, wall thickening and functional stiffening are considered predictors of cardiovascular outcomes (Morioka et al., 2021). In the case of carotids, this alteration is consistent with 10–20 years of physiological aging, but the good news for astronauts is that it is reversible upon return to Earth (Arbeille et al., 2017). The mechanisms contributing to arterial thickening in space are not fully elucidated. Since similar alterations are depicted in arteries above and below the heart, loss of pressure gradients does not offer a sufficient explanation (Patel, 2020). Rather, it is feasible that humoral factors -angiotensin II, catecholamines and insulin- are implicated in inducing endothelial dysfunction, smooth muscle cell hypertrophy and collagen deposition in the arterial wall through their phenotypic modulation from a contractile to a synthetic phenotype. In the cephalic venous system, increased jugular vein cross-sectional area and increased venous pressure were described in astronauts, often associated with stagnant flow (Kim et al., 2021).

Also radiations, both high linear-energy transfer particles and high charge and energy nuclei, play a role in shaping arterial structure in space. Earth’s atmosphere shields from cosmic radiation and solar particles, and radiation and cosmic weather are relevant hazards for the vessels. It is known that whole body irradiation is a cardiovascular risk factor, as demonstrated in survivors of atomic disasters or in patients after radiotherapy (Vernice et al., 2020). Indeed, ionizing radiations increase aortic stiffness, thicken carotid intima, promote endothelial dysfunction and accelerate atherogenesis, mainly by inducing oxidative stress and, consequently, inflammation (Patel, 2020).

**FIGURE 1:**
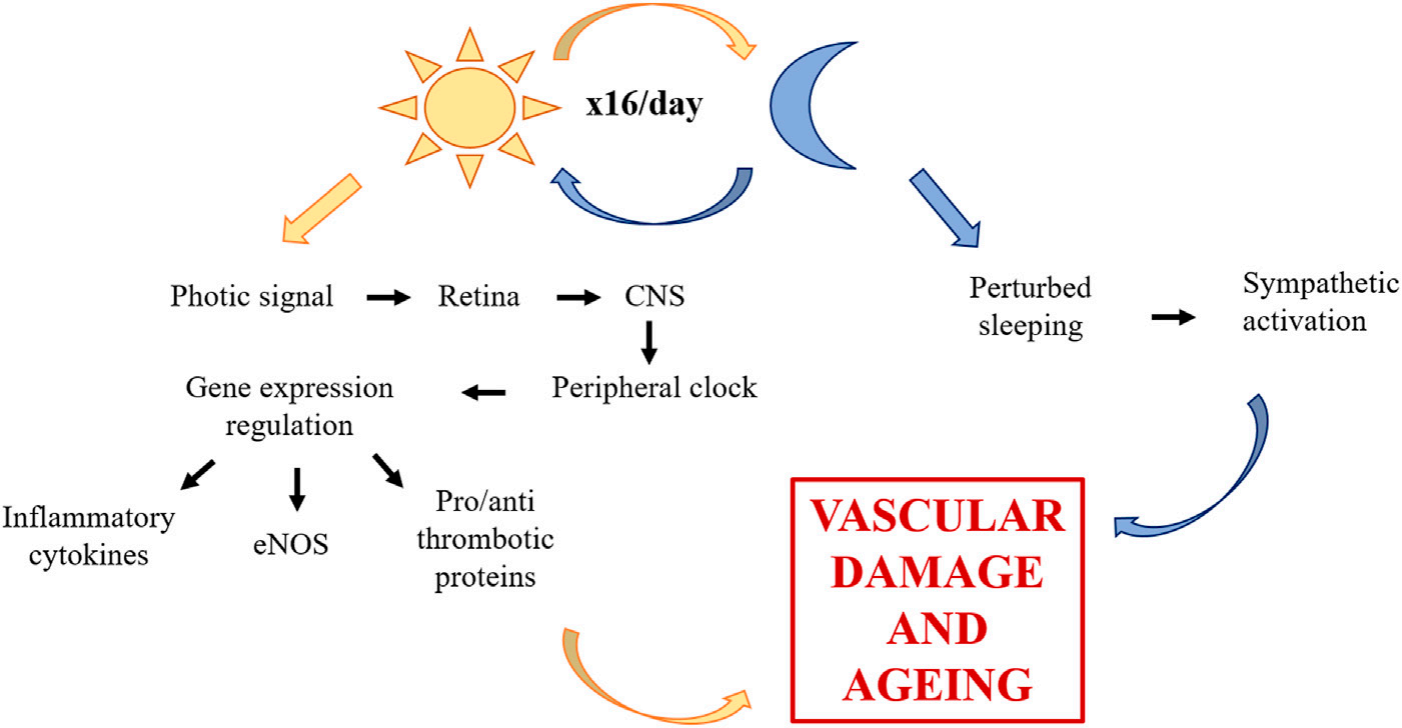
Role of circadian rhythm dysregulation on the vascular system. Through photic signal, circadian rhythm influences CNS, which then coordinates peripheral clocks of vascular cells. The alteration of eNOS, inflammatory cytokines and pro- and anti-thrombotic proteins promotes vascular damage and accelerates vascular aging. Furthermore, circadian rhythm dysregulation induces sleep disorders which increase vascular disease risk. CNS, central nervous system; eNOS, endothelial nitric oxide synthase.

Additional challenges in space are hazardous for the vessels. Life in space constrains physical activity and it well known that an active lifestyle is recommended for vascular health, since it exerts favourable effects on all cardio-metabolic risk factors (Giallauria et al., 2021). Moreover, being subjected to 16 sunrises and sunsets, astronauts undergo disruption of circadian rhythm. Because the Earth turns on its axis every 24h, life on this planet evolved taking into account circadian rhythmicity (Hughson et al., 2018), so that biological functions are aligned with environmental changes. Circadian rhythm influences an array of diverse biological processes including inflammation, redox homeostasis, metabolism, and also cardiovascular physiology (Crnko et al., 2019). The relevance of light in shaping fundamental function is due to the photic signal transmitted from the retina to the central nervous system, specifically to the hypothalamic suprachiasmatic nucleus (Man et al., 2021). The brain then coordinates peripheral clocks, which are present in each of the cardiovascular cell types and, in the end, regulate the expression of genes that control the function of vascular endothelial and smooth muscle cells, among which endothelial Nitric Oxide Synthase (eNOS), inflammatory cytokines, pro- and anti-thrombotic proteins (Crnko et al., 2019). Of note, clock misalignment promotes vascular damage and accelerates vascular aging (Chen et al., 2021; Silva et al., 2021). In addition, since light–dark cycles regulate the sleep–wake cycle and sleep disorders are common in astronauts, it should be recalled that perturbed sleeping increases vascular disease risk by impairing endothelial function and by perturbing autonomic balance in favor of sympathetic activation (Cherubini et al., 2021) (Figure 1).

**FIGURE 2:**
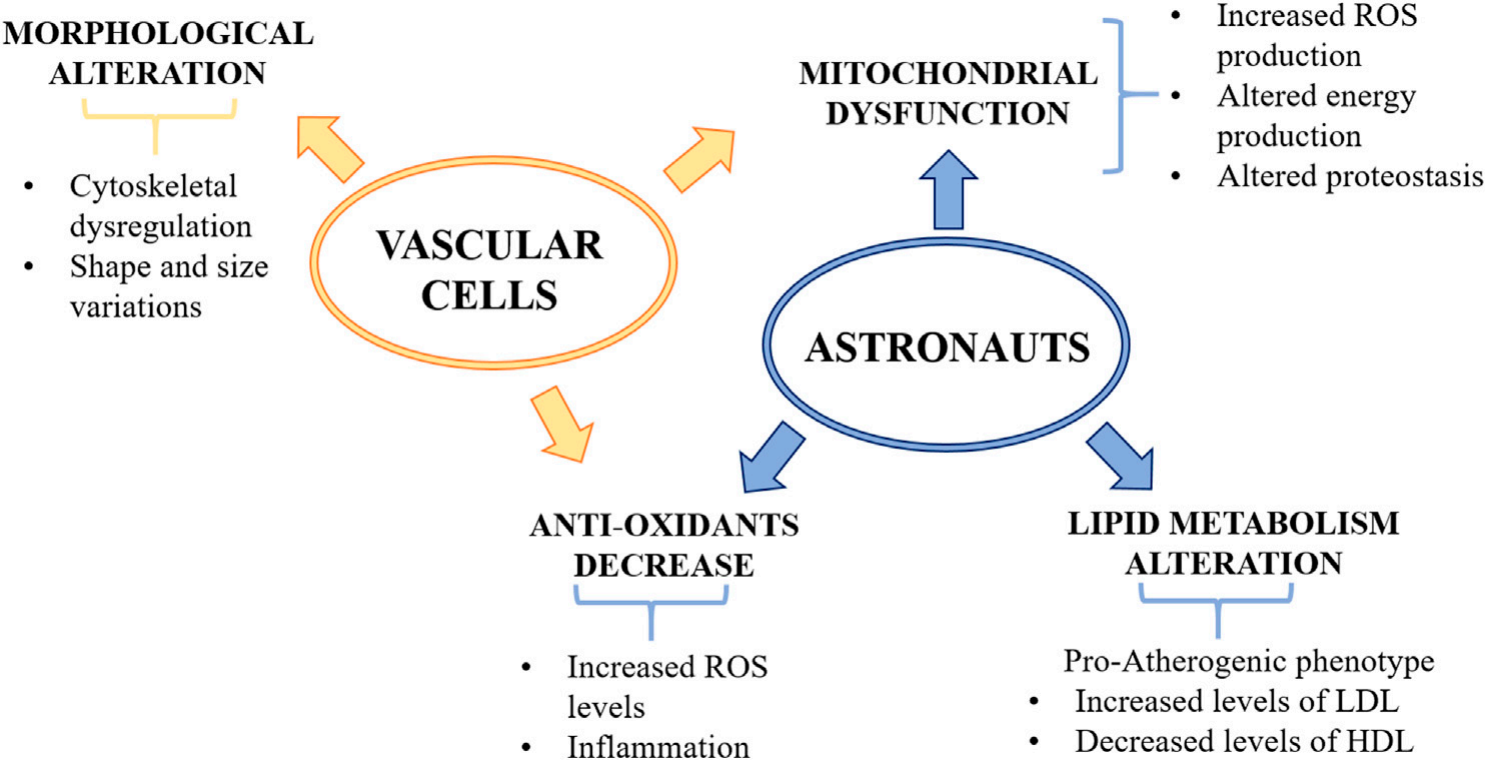
The effects of microgravity on the vascular system. Microgravity decreases anti-oxidant defenses and induces mitochondrial dysfunction, thus increasing oxidative stress and impairing energy production and proteostasis. Dysregulation of lipid metabolism was found in astronauts which showed increased level of LDL and decreased level of HDL. Vascular cells exposed to microgravity showed morphological alterations, such as cytoskeletal dysregulation and shape and size variations. ROS, reactive oxygen species; LDL, low density lipoprotein; HDL, high density lipoprotein.

Recently, medical records of fifty-nine astronauts and data derived from cells flown to space were integrated through a complex system biology analytical approach (da Silveira et al., 2020), which summarizes the effects of the various hazards and challenges occurring in space. Several issues emerge that might be relevant in explaining vascular alterations. First of all, long term space missions promote mitochondrial dysfunction, which increases oxidative stress, jeopardizes energy production and impairs proteostasis, all playing a fundamental role in vascular disease (Figure 2). Of note, anti-oxidant defenses gradually decline in astronauts and it is likely that cumulative damage by oxidative species occurs. Oxidative stress is strictly linked to inflammation and, accordingly, increased amounts of inflammatory cytokines were measured in the blood of astronauts (da Silveira et al., 2020). Spaceflight also dysregulates lipid metabolism both in humans and mice (da Silveira et al., 2020). In particular, low-density lipoproteins (LDL), which deliver cholesterol to the artery wall thus triggering atherogenesis (Borén et al., 2020), increase, whereas high-density lipoproteins (HDL), which remove cholesterol (Jomard and Osto, 2020), decrease. This lipid profile is typically considered pro-atherogenic, but it is noteworthy that LDL and HDL revert to normal levels upon return to Earth. This multi-omics analysis also highlights lower levels of vitamin D, which, apart from its traditional role in bone health, is also beneficial for the vasculature (Rosen and Taylor, 2013).

It is clear that understanding the cellular and molecular bases of vascular alterations in space relies on experiments performed *in vitro* and *in vivo* during missions or utilizing ground-based facilities. Nevertheless, it should be underscored that no simulation on Earth can mimic the complexity of the events occurring in space. As for animal models, they are helpful to explore mechanisms of acclimation as well as health implications of spaceflight. However, the interpretation of the results is complicated by the evidence that, in spite of similar body organization, animals disclose different developmental times and different lifespans (Matsuda et al., 2020). Accordingly, modulation of gene expression and biochemical reactions require much longer times in humans than in mice (Matsuda et al., 2020).

Although data are fragmentary, sometimes conflicting and far from being complete, experimental studies indicate that mechanical forces, radiations and a large array of biochemical factors cooperate to model the vessel wall.

## Space Stresses Vascular Cells: Suggestions From *in vivo* Studies

Rodents have been widely utilized as a model organism to get insights into human physiology and pathophysiology. This applies also to space-related research. In the NASA rodent research-1 mission which lasted up to 37 days, mice are initially disoriented, but after a few days they begin to move in circle, an event that has been interpreted as a potential stereotyped behavior to fight anxiety (Ronca et al., 2019). The increased physical activity might also explain why muscle atrophy is less marked than expected (Choi et al., 2020), and might play beneficial actions on the vasculature, also by reducing the neuro-endocrine alterations typically associated with stress. Of interest, transcriptomics and proteomics showed disruption of lipid metabolism in the liver of spaceflown mice vs. their ground controls (Beheshti et al., 2019a), which might account for the altered lipid profile in the blood (da Silveira et al., 2020), eventually promoting arterial damage. From their multi-omics analysis, the authors also highlight alterations of the circadian clock pathway, as described in humans. Focusing on the vascular system, it was shown in mice that 13 days onboard the shuttle diminished myogenic vasoconstrictor tone and vascular stiffness, and increased the maximal diameter of cerebral arteries without any change in medial wall thickness. As a consequence, cerebral perfusion was elevated during spaceflight (Taylor et al., 2013), similar to reports in astronauts.

**FIGURE 3:**
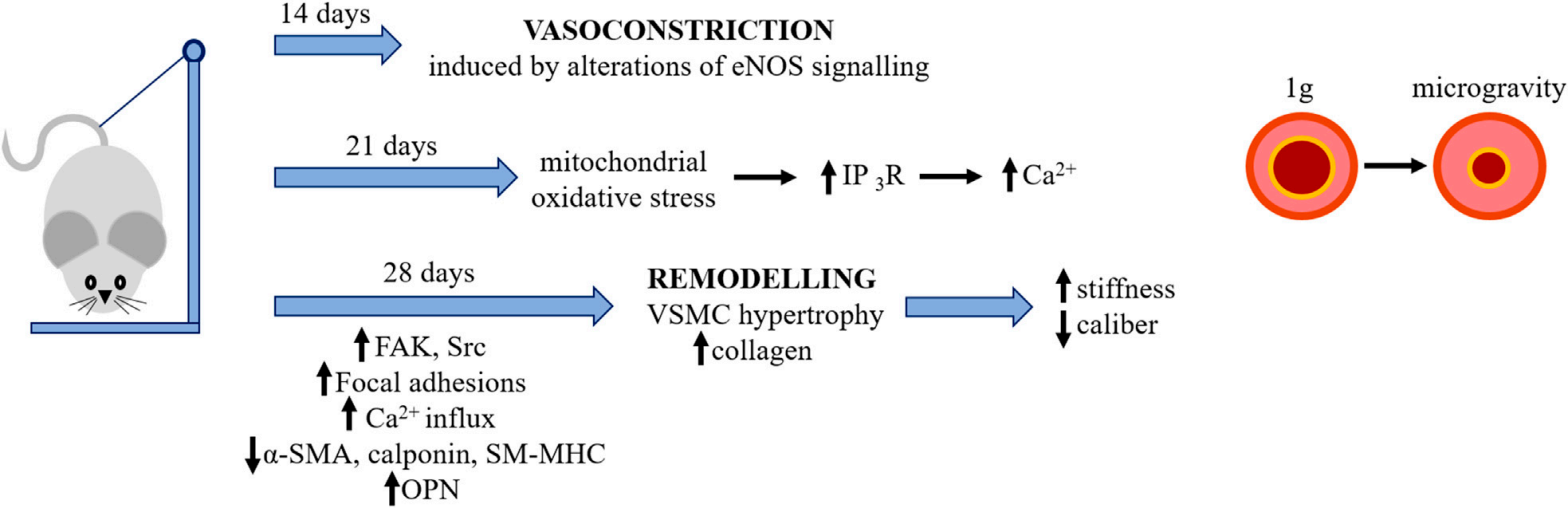
The effects of simulated microgravity on rodents’ cerebral arteries. Microgravity was simulated by HU (see text). Simulated microgravity induces initially vasoconstriction and then remodelling of rodents’ cerebral arteries. IP_3_-R, inositol 1,4,5-trisphosphate receptor; FAK, focal adhesion kinase; α-SMA, alpha-smooth muscle actin; SM-MHC, smooth muscle-myosin heavy chain; OPN, osteopontin.

However, at the moment data on the effects of spaceflight on the vessels are scant. Some hints are derived from experiments with hindlimb suspension (HU) rodents. HU is a spaceflight analog which recreates the fluid shift from the lower extremities towards the upper part of body, thus mimicking some effects of microgravity. It should be underscored that region-specific vascular remodeling has been reported in HU rats. In an attempt to describe vascular alterations in HU rodents, we propose the following time line of events (Figure 3). After 2 weeks HU promotes cerebral artery vasoconstriction mainly through alterations in eNOS signaling (Wilkerson et al., 2005). The high pressure-induced mechanical stretch seems to be the driving force for the de-differentiation of cerebral vascular smooth muscle cells (vSMC). After 21 days the content of cytoplasmic Ca^2+^ in cerebral arteries is increased since Ca^2+^ is released in a inositol 1,4,5-trisphosphate receptor (IP_3_-R) dependent manner, and event that can be prevented by the mitochondrial antioxidant MitoTEMPO (Liu et al., 2021). After 28 days (Gao and Chilibeck, 2020), clear structural alterations of the arteries are described. Whereas reduced myogenic tone is reported in the vessels of the lower limb and splanchnic district, high vaso-reactivity and hypertrophy are observed in the large arteries above the hearth (Looft-Wilson and Gisolfi, 2000). At this time point, rat cerebral arteries display lower amounts of α-smooth muscle actin (α-SMA), calponin and smooth muscle-myosin heavy chain (SM-MHC), and higher levels of osteopontin (OPN), indicating the transition from a contractile to a synthetic phenotype. This is triggered by mitochondrial oxidative damage which activates the PKR-like endoplasmic reticulum (ER) kinase (PERK)/C/EBP-homologous protein (CHOP) pathway and is attenuated by the administration of MitoTEMPO (Zhang R et al., 2020). Accordingly, cerebrovascular smooth muscle cells isolated from the arteries of these rats downregulate contractile markers and upregulate synthetic markers (Jiang et al., 2018; Zhang B et al., 2020), events mediated by mechanotransducers such as focal adhesions and calcium channels (Jiang et al., 2018; Zhang B et al., 2020). In the basilar artery of HU rats, smooth muscle cells are hypertrophic and the extracellular deposition of collagen is increased in association with an increased number of focal adhesions and higher expression of focal adhesion kinase (FAK) and Src, while the opposite occurs in the femoral artery (Jiang et al., 2018). Moreover, increased blood pressure and mechanical stretch lead to Ca^2+^ influx into vSMC and, consequently, to high resting cytosolic Ca^2+^, which is a feature of de-differentiated vSMC (Matchkov et al., 2012). Interestingly, vSMC isolated from 28 days HU rat upregulate the T type Cav3.1 channel, which increases Ca^2+^ entry, through the downregulation of miR-137. The inhibition of the T type Cav3.1 channel as well as the overexpression of miR-137 prevent vSMC de-differentiation by controlling the calcineurin/NFATc3 pathway (Zhang B et al., 2020). In the aorta of HU rats, stiffness results from the increased content of collagen and cross-linking activity of lysyl oxidase and transglutaminase (Tuday et al., 2009). These results are in contrast with data obtained in space. This inconsistency can be ascribed to the fact that other extraterrestrial factors, such as radiations which are considerably higher aboard the International Space Station (ISS) than on Earth, might be implicated. The few results available point to radiation as crucial players in vascular remodeling. Indeed, in rats, simulated space irradiation with high energy iron-ion radiations impaired endothelium-dependent vasodilation of the aorta and increased aortic stiffness because of the accumulation of reactive oxygen species and the decrease of nitric oxide (NO) (Soucy et al., 2011). Moreover, iron-ion radiation accelerates atherogenesis in apo-E deficient mice (Yu et al., 2011). An interesting study on mice evaluates the single and combined effects of simulated space irradiation and weightlessness obtained by HU and shows their synergic action in impairing endothelium dependent vasodilatation of muscle resistance arteries through the induction of oxidative stress (Ghosh et al., 2016). The long-term effects of weightlessness and simulated space radiation on vessels was investigated after 6 months recovery. The study shows that, while endothelial function is recovered in the HU mice, impairment of endothelium-dependent vasodilation endured in the irradiated mice because of the persistent alteration of the eNOS pathway and elevated oxidative stress (Delp et al., 2016).

Another relevant topic relates to the effects of altered circadian rhythms on the vasculature. It has long been known in rodents that aberrant circadian rhythms determine pathological remodeling and vascular damage (Anea et al., 2009). The analysis of eight RNA-seq datasets from NASA GeneLab on mice subjected to 37-day spaceflight mission demonstrates alteration of clock gene expressions in different tissues compared to ground control mice. Since glucocorticoids (GC) modulate the expression of clock genes via GC response elements, the increase of plasma and urine cortisol levels observed in spaceflown mice might contribute to the perturbation of the circadian rhythm (Fujita et al., 2020; Cahill et al., 2021). It will be intriguing to broaden these studies to investigate the modulation of factors involved in vascular dysfunction in spaceflown rodents.

## Space Stresses Vascular Cells: Suggestions From *in vitro* Studies

Cell culture experiments represent a valuable complement to *in vivo* models to get insights into cellular functions and the underlying molecular mechanisms. Experiments in real microgravity are the gold standard to really appreciate what happens to cells during spaceflight, combining the effects of microgravity with other extraterrestrial players, such as space radiations. Focusing on vascular cells, we will initially summarize what happens in space to vSMC, quiescent contractile cells located in the tunica media. In response to changes of haemodynamic conditions and/or to the presence of different soluble mediators, vSMC can acquire a proliferative and biosynthetic phenotype. Only two experiments were performed in space, one on the ISS and the other onboard the shuttle, both for 8 days using rat vSMC. The cells maintain their viability and contractile phenotype, and downregulate ryanodine receptor R1, a reticulum calcium channel, which inhibits L-type channels, thus leading to relaxation (Dabertrand et al., 2012).

Because experiments in space are expensive and subjected to several restraints, bioreactors that simulate some aspects of spaceflight are commonly used (Maier et al., 2015; Baran et al., 2022). The space biology community has mainly investigated the effects of simulated microgravity on cell functions, and far less is known about the effects of radiations. In accordance with data obtained in space (Dabertrand et al., 2012), experiments performed in simulated microgravity showed that vSMC upregulate stress proteins, remain quiescent and maintain their contractile phenotype (Coinu et al., 2006; Kang et al., 2013), thereby underscoring that mechanical unloading has a role in shaping vSMC behavior. However, also radiations and increased amounts of stress and inflammatory mediators, reactive oxygen species (ROS), insulin and angiotensin II –as described in astronauts (see above)- should be taken into account. As an example, high angiotensin II triggers oxidative stress, mitochondrial dysfunction, and promotes vSMC senescence (Okuno et al., 2020), beyond sensitizing the cells to the detrimental effects of catecholamines. Ionizing radiations change vSMC phenotype (Heckenkamp et al., 2004) and increase the sensitivity of their myofilaments to Ca^2+^ through the activation of protein kinase C (Soloviev et al., 2005). Therefore, it is likely that biochemical stimuli, radiations and mechanical unloading all contribute to carve vSMC behavior, thereby leading to arterial stiffness and tunica media thickening.

More attention has been devoted to the endothelium, which is located at the interface between the blood and the tissues and is responsible for vascular integrity, thus guaranteeing an adequate blood supply to all the tissues (Krüger-Genge et al., 2019). To exert their multiple vital functions, endothelial cells continuously integrate biochemical and mechanical signals arising from the microenvironment (Krüger-Genge et al., 2019). In particular, EC are very sensitive to microgravity as demonstrated by the numerous experiments performed both in space and simulated gravity on human micro and macro-vascular EC (recently reviewed by Morbidelli et al., 2021; Baran et al., 2022). It should be recalled that both spaceflight and ground-based microgravity analogues are considered as low-shear stress environments (Delp et al., 2000; Zhang, 2001; Hughson et al., 2018) and this influences endothelial behavior. Accordingly, EC are affected by the reduction of a physiological shear (Inglebert et al., 2020; Locatelli et al., 2022), which primarily shapes cell morphology and cytoskeletal organization.

Human umbilical vein EC (HUVEC) have been extensively used as a model of macrovascular EC to advance our knowledge about the physiology and pathophysiology of the endothelium, and here we will limit our discussion to the common behavioral features emerging from studies on HUVEC cultured in space and on Earth using various space analogues. Spaceflight alters HUVEC shape and size, disorganizes the cytoskeleton and reduces mitochondrial mass (Kapitonova et al., 2012), a bulk of alterations confirmed in HUVEC exposed to simulated microgravity on Earth (Locatelli et al., 2020; Locatelli and Maier, 2021). Cytoskeletal remodeling, which occurs very rapidly after exposure to microgravity, seems to trigger endothelial adaptation to microgravity (Locatelli and Maier, 2021) being involved in the activation of stress response and alterations of mitochondrial content. Microgravity-induced stress response is complex and dynamic (Versari et al., 2013; Cazzaniga et al., 2019). The Sphinx experiment run in space for 10 days on HUVEC demonstrated the modulation of more than 1,000 genes (Versari et al., 2013), the most overexpressed of which is Thioredoxin Interacting Protein (TXNIP). TXNIP is also markedly upregulated both at the RNA and protein level in HUVEC exposed to simulated microgravity for 10 days. Thanks to the possibility of performing experiments in kinetics in simulated microgravity, it was demonstrated that the sequential upregulation of various stress proteins contrasts oxidative stress, prevents apoptosis and establishes a novel homeostasis that preserves cell viability (Cazzaniga et al., 2019). All existing evidences point to a role of microgravity alone in modulating endothelial function. However, since EC are the most radiosensitive component of the vascular wall (Soloviev and Kizub, 2019), also the role of radiations should be considered, and this might explain some inconsistencies with results from spaceflown HUVEC in terms of oxidative stress and inflammation. To this purpose, it should be recalled that the first effect of radiation is the rapid generation of free radicals because of H_2_O radiolysis, followed by radiation-mediated activation of sphingomyelinase and some pro-oxidant enzymes which maintain high the levels of reactive oxygen species (Soloviev and Kizub, 2019). This evokes endothelial dysfunction, the first step in atherogenesis. Notably, by utilizing GeneLab datasets, common molecular pathways between simulated space radiation and HUVEC flown on the ISS were identified (Beheshti et al., 2019b). Therefore, we propose that it is the combination of microgravity and radiations that provokes oxidative stress in spaceflown HUVEC. Moreover, the increases of epinephrine, insulin, angiotensin II and LDL, as detected in astronauts, favor inflammation and accelerate atherogenesis (Sheng and Zhu, 2018), and similar effects are exerted on EC by alterations of the circadian clock pathways (Paschos and FitzGerald, 2010; Navar, 2014; Oyama et al., 2019).

We now turn to human microvascular EC (MEC), which represent the majority of the overall endothelial surface and are implicated in innate and adaptive immune response (Danese et al., 2007). In MEC more than 2,500 genes are modulated in space, and the response to the bacterial endotoxin lipopolysaccharide is impaired. This study highlights significant metabolic perturbations and links energy production to impaired immune function in spaceflown MEC (Chakraborty et al., 2018). Accordingly, an altered energy profile was reported in MEC cultured on the ISS (Barravecchia et al., 2021). This study shows that spaceflight stresses MEC as demonstrated by alteration in cell shape, size and volume due to the massive cytoskeletal rearrangement, altered distribution and morphology of mitochondria, DNA damage and telomere erosion. Simplistically, it can be concluded that space promotes a senescent phenotype in MEC. The novelty of this work is the dissection between the effects of radiations and microgravity in shaping MEC behavior. Some alterations of MEC in space, such as oxidative stress, inflammation and DNA damage, can be mainly ascribed to the effects of radiations, in agreement with previous results showing that MEC exposed to field gamma and proton radiation alone or in combination activate the inflammasome pathway through oxidative stress (Chatterjee et al., 2019). In general, it is clear that experiments performed on Earth not only mimic some events observed in space, but also allow to identify the molecular pathways involved, a knowledge essential to develop targeted countermeasures.

EC are actively involved in regulating vascular tone and maintaining blood fluidity, and NO plays a role in both the events. Indeed, NO is the most powerful endogenous vasodilator and also inhibits platelets adhesion to the endothelium (Zhao et al., 2015). Therefore, the production of NO in EC cultured in simulated microgravity has attracted a lot of attention, since NO dysregulation might offer insights into vascular alterations in space as well as into orthostatic hypotension experienced by the majority of astronauts after returning to Earth. While no data are available from EC cultured in space, HUVEC and human MEC as well as endothelial progenitors exposed to simulated microgravity produce and release more NO than controls through the upregulation of eNOS (Versari et al., 2007; Mariotti and Maier, 2008; Kong et al., 2021). eNOS activity is increased as a consequence of cytoskeletal changes, which impair transport of caveolins to caveolae (Grenon et al., 2013; Shi et al., 2016). It is noteworthy that also ionizing radiation activate eNOS in HUVEC through the up-regulation of protein kinase C (PKC)-β and the increase of ROS (Sakata et al., 2015), while angiotensin II exerts an opposite effect (Ding et al., 2020). Only experiments in spaceflown cells will provide a definitive answer. A last topic to mention is about coagulation. Under normal conditions, EC provide an anti-aggregant and anti-coagulant surface to the blood. However, alterations of flow, inflammation, oxidative stress or any harm to EC promote the acquisition of a pro-coagulant phenotype. A left internal jugular venous thrombosis was reported in an astronaut (Auñón-Chancellor et al., 2020), thus suggesting that spaceflight might promote coagulation. This might be due to venous stasis in the cephalic venous districts, which facilitates platelets’ interaction with the vessel wall and damages EC through dysregulated cytokine network and free radical synthesis (Kim et al., 2021). Also altered platelets’ function might be implicated, but very little is available on this issue in the literature (Locatelli et al., 2021).

Obviously, translating data from cultured cells into prediction of what happens in humans is very complex.

It should be kept in mind that “spaceflight effects were more evident in isolated cells than whole organs, suggesting that tissue complexity plays an essential role in response to space-related stress” (da Silveira et al., 2020).

## From Knowledge to Prevention: What can Be Done?

Space exploration entails several health challenges, including vascular risk (Navasiolava et al., 2020). Delp’s study suggests that lunar astronauts, the only ones who travelled in deep space, show higher cardiovascular risk that non flight or low orbit astronauts (Delp et al., 2016). This difference is mainly ascribed to the fact that only lunar astronauts travelled outside the Earth’s magnetosphere, which diverts radiations. Since a short duration stay in deep space is detrimental to the vasculature, specific strategies, including novel radiation shielding materials, should be developed in view of long term missions and permanent space colonization.

Different countermeasures have been proposed to protect vascular function during spaceflight and prevent post-flight orthostatic intolerance. The reproduction of artificial gravity would represent the gold standard. To the best of our knowledge, only one study was performed in space and showed that housing mice in a centrifuge for 35 days mitigates space-induced apoptosis in retinal endothelial cells (Mao et al., 2018). However, a procedure based on human centrifugation is unrealistic, and alternative strategies are foreseen.

Regular exercise is associated with metabolic and cardiovascular adaptations which improve general health, including vascular function (Boeno et al., 2019). Accordingly, chronic physical constraint obtained by bed rest exacerbates vascular, and in particular, endothelial dysfunction (Pedrinolla et al., 2020). Indeed, physical training, and especially aerobic exercise, improves intracellular redox balance and mitochondrial health, reduces the levels of systemic inflammatory markers, ameliorates endothelial function and also modulates the circadian clocks thus resynchronizing vascular clock misalignment (Silva et al., 2021). On the contrary, the effects of resistive exercise on vascular function are more controversial, since it seems to have deleterious effects on endothelial functions, probably for the sustained elevation in blood pressure (Morishima et al., 2018). However, the combination of resistive and aerobic exercise has been demonstrated to ameliorate endothelium dependent vasodilation and preserve the number of circulating EC (Maiorana et al., 2000; Demiot et al., 2007). It should be recalled that the daily routine of an astronaut includes two hours of physical exercise to prevent bone and muscle loss, with positive impacts on the vasculature. Many possibilities are available and it is feasible that customized personal exercise programs should be envisioned on the basis of individual differences, including gender difference, to maintain physical fitness. Among others, high-intensity interval training (HIIT), which comprises short sessions of maximal-intensity exercise alternated with less intense recovery intervals, might be effective, since it ameliorates endothelial function and reduces arterial stiffness (Hurst et al., 2019). Another valid countermeasure is Whole Body Vibration (WBV), which is reported to decrease arterial stiffness (Otsuki et al., 2008) and exert beneficial effects on bone mass (Gómez-Cabello et al., 2012), neuromuscular function (Fontana et al., 2005), and the endocrine system (Di Loreto et al., 2004). Also the use of Low Body Negative Pressure (LBNP) might be of interest. In bed rest studies LBNP ameliorates orthostatic hypotension by maintaining the vasoconstriction response, prevents endothelial dysfunction and counteracts headward fluid shift when combined with fluid loading (i.e., salt and water) and nutritional supplementation (Marshall-Goebel et al., 2019). A similar technique, called Blood Flow Restriction (BFR), which consists in the application of a local external pressure to the limbs to create partial restriction of blood flow (Willis et al., 2019), seems to be useful to prevent not only sarcopenia (Patterson et al., 2019), but also orthostatic intolerance. Moreover, the combination of BFR and HIIT stimulates vascular response (Willis et al., 2019).

**FIGURE 4:**
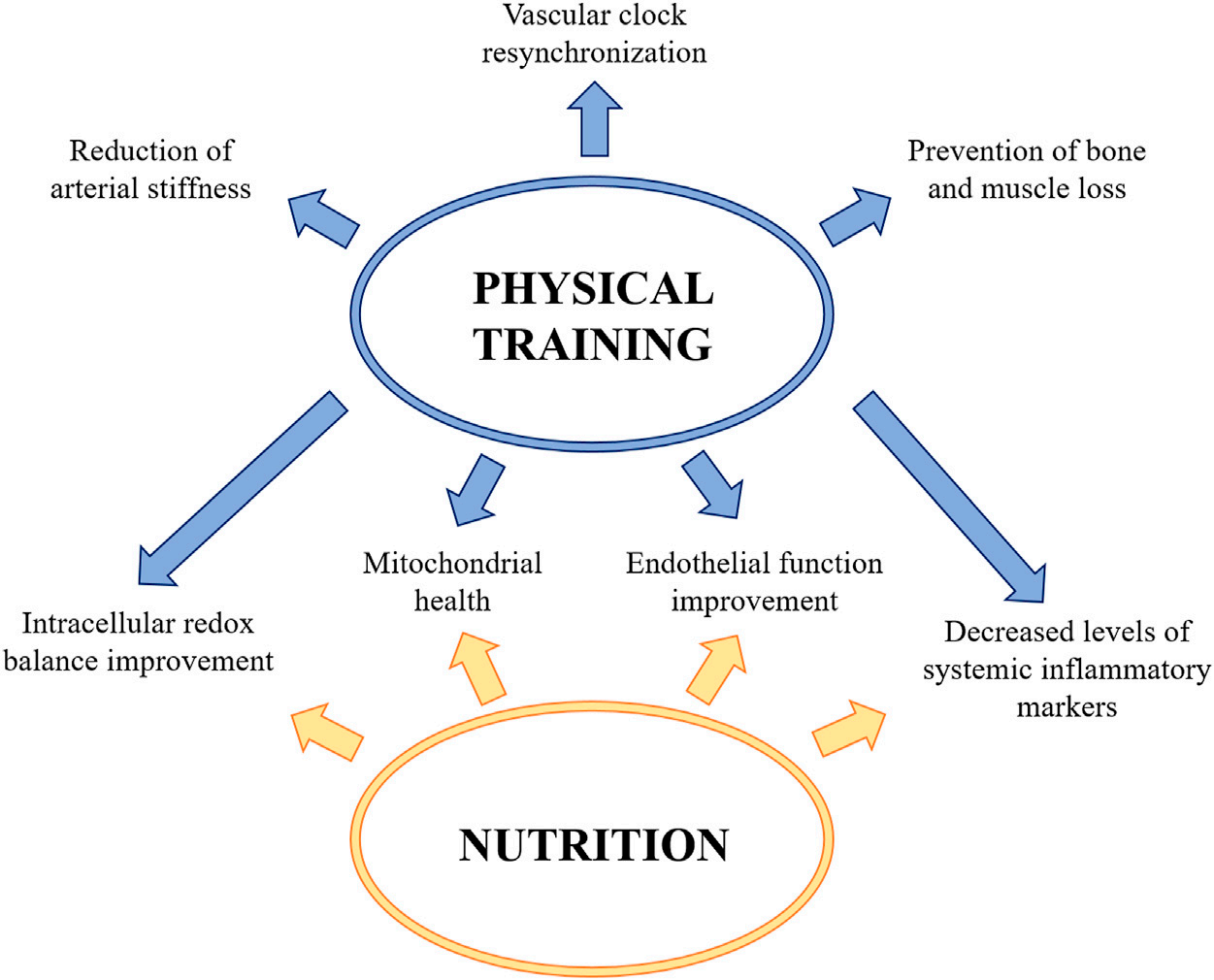
Countermeasures to protect vascular function during spaceflight and prevent post-flight orthostatic intolerance. Physical training and a correct and adequate nutrition improve intracellular redox balance and mitochondrial health, reduce the levels of systemic inflammatory markers and ameliorate endothelial function. Physical training also contributes to the arterial stiffness reduction, to prevent the bone and muscle loss and to resynchronize vascular clock.

Much effort has been made to investigate potential effect of nutrition as an effective countermeasure. The ideal diet might provide the adequate fuels to the tissues and at the same time minimize oxidative stress, insulin resistance and inflammation (Zwart et al., 2021). Astronauts on the ISS are supplied with rehydratable, thermostabilized, natural and irradiated food products which usually are composed by 80% standard set of food containers and 20% “preference containers” chosen by the crew. Every 2–4 months the ISS is supplied with some fresh fruits and vegetables and some semi-shelf-stable specialty items. A first issue to consider is that nutrition is negatively affected by space because of several factors, including motion sickness, altered circadian rhythms, confinement and low palatability of food (Smith et al., 2005). The outcome is that astronauts do not achieve their required energy intake (Costa et al., 2021), mainly because of reduced appetite due, in part, to increased amounts of the satiety hormone Glucagon-like peptide-1 (Bergouignan et al., 2016). Since decreased caloric and inadequate intake of micronutrients generates inflammation and oxidative stress (Ames, 2006), it is necessary to design balanced diets and appropriate supplementation with functional foods and antioxidant micronutrients. However, strategies to counteract the detrimental effects of increased free radicals by supplementing an anti-oxidative cocktail in manned missions have been unsuccessful (Meerman et al., 2021). This disappointing result might be due to the altered intestinal absorption reported in space (Yang et al., 2020) and to inter-individual variability, an issue that might be overcome by a personalized approach. Also targeting the gut microbiome, which is linked to food intake, with specifically designed probiotics or dietary supplements, could be useful, also in the light of an altered composition and function of the microbiome in space (Turroni et al., 2020; Siddiqui et al., 2021). Furthermore, gut microbes seem to control intestinal release of satiety hormones and directly stimulate central appetite pathways (Covasa et al., 2019) (Figure 4).

## Conclusion

Astronauts will face multiple hazards simultaneously during long duration missions outside of the low earth orbit. Therefore, it is imperative to consider the combined effects of these hazards to define health risk and tailor adequate countermeasures. Some inconsistencies emerge when comparing data obtained in space with those generated using simulations on Earth. This is not surprising because no simulation can include all the extraterrestrial factors. There are many open questions that await an answer and several issues that need to be untangled. However, many experiments are ongoing and many more are expected to grant a safe future in space.
